# Metanetwork Transmission Model for Predicting a Malaria-Control Strategy

**DOI:** 10.3389/fgene.2018.00446

**Published:** 2018-10-17

**Authors:** Bo Li, Xiao Liu, Wen-Juan Wang, Feng Zhao, Zhi-Yong An, Hai Zhao

**Affiliations:** ^1^Shandong Technology and Business University, School of Computer Science and Technology, Yantai, China; ^2^Shandong Co-Innovation Center of Future Intelligent Computing, Yantai, China; ^3^Northeastern University, School of Computer Science and Engineering, Shenyang, China; ^4^Yantai Yuhuangding Hospital of Qingdao University, Reproduction Medical Center, Yantai, China

**Keywords:** malaria, metanetwork, transmission model, centrality measure, refractory mosquito

## Abstract

**Background:** Mosquitoes are the primary vectors responsible for malaria transmission to humans, with numerous experiments having been conducted to aid in the control of malaria transmission. One of the main approaches aims to develop malaria parasite resistance within the mosquito population by introducing a resistance (R) allele. However, when considering this approach, some critical factors, such as the life of the mosquito, female mosquito fertility capacity, and human and mosquito mobility, have not been considered. Thus, an understanding of how mosquitoes and humans affect disease dynamics is needed to better inform malaria control policymaking.

**Methods:** In this study, a method was proposed to create a metanetwork on the basis of the geographic maps of Gambia, and a model was constructed to simulate evolution within a mixed population, with factors such as birth, death, reproduction, biting, infection, incubation, recovery, and transmission between populations considered in the network metrics. First, the same number of refractory mosquitoes (RR genotype) was introduced into each population, and the prevalence of the R allele (the ratio of resistant alleles to all alleles) and malaria were examined. In addition, a series of simulations were performed to evaluate two different deployment strategies for the reduction of the prevalence of malaria. The R allele and malaria prevalence were calculated for both the strategies, with 10,000 refractory mosquitoes deployed into randomly selected populations or selection based on nodes with top-betweenness values. The 10,000 mosquitoes were deployed among 1, 5, 10, 20, or 40 populations.

**Results:** The simulations in this paper showed that a higher RR genotype (resistant-resistant genes) ratio leads to a higher R allele prevalence and lowers malaria prevalence. Considering the cost of deployment, the simulation was performed with 10,000 refractory mosquitoes deployed among 1 or 5 populations, but this approach did not reduce the original malaria prevalence. Thus, instead, the 10,000 refractory mosquitoes were distributed among 10, 20, or 40 populations and were shown to effectively reduce the original malaria prevalence. Thus, deployment among a relatively small fraction of central nodes can offer an effective strategy to reduce malaria.

**Conclusion:** The standard network centrality measure is suitable for planning the deployment of refractory mosquitoes.

**Importance:** Malaria is an infectious disease that is caused by a plasmodial parasite, and some control strategies have focused on genetically modifying the mosquitoes. This work aims to create a model that takes into account mosquito development and malaria transmission among the population and how these factors influence disease dynamics so as to better inform malaria-control policymaking.

## Introduction

Malaria is a mosquito-borne infectious disease that is caused by a parasitic plasmodium. Attempts to control malaria transmission are expensive and unsustainable, with many African countries experiencing a major economic burden due to costs exceeding $10 billion each year (The Earth Institute of Columbia University, 2001; World Health Organization, 2018). Numerous attempts have been made to control malaria transmission. One approach is to use water-soluble phosphine complexes that have been shown to be effective during plasmodial early sporogonic stages *in vitro*, which is the sexual form responsible for infecting the mosquito vector (Tapanelli et al., 2017). In another study, a relatively new antimalarial compound, tafenoquine (TQ), was combined with a low dose of artesunate (ATN), and its effect on avian *Plasmodium gallinaceum* was examined in *Aedes aegypti* (Tasai et al., 2017). Blood-stage multiplication, gametocyte development, and transmission were examined, and the results showed that TQ, when combined with a low dose of ATN, is effective for limiting avian malaria transmission and offers a safe and effective treatment. In a study attempting to monitor parasite transmission in the field, a novel one-step reverse transcriptase real-time polymerase chain reaction (PCR) (direct RT-PCR) was used to detect *Plasmodium falciparum* by amplifying the RNA targets directly from the blood samples and developed to identify gametocyte-specific transcripts (Taylor et al., 2017). There are also many other tactics that have been used to combat malaria, including the following: patient treatment with artemether–lumefantrine, which is effective against uncomplicated malaria (Teklemariam et al., 2017); the development of a Pfs48/45-based transmission-blocking malaria vaccine (Theisen et al., 2017); rapid diagnostic tests (RDTs) to assess the presence of sub-RDT Plasmodium falciparum as well as of Borrelia, Coxiella burnetii, and Babesia applying molecular tools (Toure et al., 2017); gaining a better understanding of the relation between deforestation and malaria transmission (Tucker et al., 2017); developing a transmission model based on the temperature-dependent incubation period (Wang and Zhao, 2017); developing laboratory quality assurance for malaria diagnostics (Wanja et al., 2017); assessing malaria transmission risk along the Thailand–Myanmar border (Ya-umphan et al., 2017); and the functional characterization of *Plasmodium berghei* PSOP25 during ookinete development and as a malaria transmission-blocking vaccine candidate (Zheng et al., 2017).

Recently, multiple research groups have attempted to tackle malaria and other related mosquito-borne diseases by attempting to either eliminate mosquito populations or introduce malaria parasite resistance (Scott et al., 2002). When attempting to eliminate mosquito populations, two different approaches are employed, including the release of lab-grown infertile male mosquitoes or female mosquitoes that only give birth to male offspring (OXITEC, 2018). However, how the refractoriness gene will affect the spreading of the malaria is not clear. Hence, understanding how the mosquitoes will affect the disease dynamics once a potential solution is released into the environment is important. Furthermore, a complex network can serve as an effective tool for modeling social-spreading behavior between populations on the basis of the topology of the network (Small and Tse, 2010; Telesford et al., 2011; Rothkegel et al., 2012; Yang et al., 2013; Caravaca et al., 2014; Ozkanlar and Clark, 2014) and providing a graph that contains nontrivial structural features (Gasparri et al., 2009; Steele Gray et al., 2016; Steinig et al., 2016; Benitez et al., 2017). However, studies focused on malaria transmission have been limited (Kiang et al., 2006; Liu et al., 2012; Aleksejs et al., 2015; Wilson et al., 2015) and have not considered some critical factors, such as the life of a mosquito, fertility capacity of female mosquitoes, the differential equation transmission model, or further transmission to other populations. In this study, a complex network model was constructed on the basis of two different aspects: evolution within mosquito and human populations (specifically examining biting, breeding, and infection) and transmission between populations (mosquito and human migration based on topology). The model was also based on time steps (one or more days, one day in this research project).

## Methods

### Defining subjects

A person who has not been bitten or has been bitten, but not infected, or is an incubation carrier was defined as being malaria-negative. A person who had been bitten by a mosquito and had become infected was defined as being malaria-positive. A mosquito that is fully susceptible to malaria was defined as being homozygous susceptible (S), with a genotype of SS; while a susceptible phenotype can also be seen in heterozygotes carrying the resistant (R) allele, with a genotype of RS. Female mosquitoes with an SS or RS genotype will have a certain probability of becoming infected after biting a malaria-positive human, whereas female mosquitoes possessing an RR genotype will not be infected by malaria. Since male mosquitoes do not bite humans, they would not be infected by malaria. This would also be true for larvae that do not bite, reproduce, or migrate.

### Understanding evolution within the populations

Understanding evolution within a mosquito population involves examining biting, breeding, and infection, and is based on different subtypes of mosquitoes and humans. In mosquitoes, gene selection is driven by natural evolution; thus, this process needs to be established. However, the time scale in humans is far larger than that in mosquitoes; thus human evolution (reproduction and death) is not taken into account.

### Larva

A fraction of the larvae is assumed to be removed from the mosquito population before reaching adulthood, with elimination occurring within the first 11 days. Larval survival depends on fitness (Orr, 2009), with genotypes that are associated with partial or full malaria refractoriness (RS or RR) being advantageous for females when malaria is prevalent and being disadvantageous otherwise; male larvae are assumed to be unaffected. Potential larval genotypes included SS, RS, or RR, with adult mosquito genotypes described in the “Adult mosquito” section. The following fitness formulas were used as previously described (Boëte and Koella, 2002):

(1) $$\begin{array}{l} w_{f,ss\;}=d\cdot \;\left(1-\exp\left(-\gamma \cdot \;\left(k+\epsilon \right)\right)\right)\cdot \alpha \end{array}$$

(2) $$\begin{array}{l} {\text{w}}_{\text{f},\text{rs }}=\text{d}\cdot \;\left(1-\exp\;\left(-\gamma \cdot \;\left(\text{k}+\epsilon \right)\cdot \;\left(1-\text{h}\cdot \text{s}\right)\right)\right)\cdot \alpha \end{array}$$

(3) $$\begin{array}{l} w_{f,rr\;}=d\cdot \;\left(1-\exp\;\left(-\gamma \cdot \;\left(k+\epsilon \right)\cdot \;\left(1-s\right)\right)\right)\cdot \alpha \end{array}$$

(4) $$\begin{array}{l} w_{m,ss\;}=w_{m,rs}\;={\;w}_{m,rr}=d \end{array}$$

(5) $$\begin{array}{l} \gamma =\frac{N_{h,pos}}{N_{h,total}}, \end{array}$$

where *w* is the fitness for a given gender and genotype (values between 0 and 0.2), *d* is the larval death rate (default value: 0.2), γ is the prevalence of malaria within the human population (value: 0–1), *k* is the rate of mosquitoes biting humans (default value: 0.9), *s* is the refractoriness (default value: 0.9, i.e., resistance to malaria), *h* is dominance (default value: 1), α is parasite virulence (default value: 0.3, i.e., loss of fitness due to parasite), and ϵ is a tune parameter (default value: < 0.001) that regulates *w* if there is no malaria. The fitness values (*w*_*f, ss*_: 0–0.04; *w*_*f, rs*_: 0–0.01; *w*_*f, rr*_:0–0.01) were then used to calculate the number of new mosquitoes for a given genotype at each time step:

(6) $$\begin{array}{l} N_{l_{t}}=N_{l_{t-1}}+{\;N}_{nl_{t}}\left(1-w\right), \end{array}$$

where, *N*_*l*_*t*__ is the number of larvae at the current time step (see equation 8).

### Adult mosquitoes

A fraction of the adults is also assumed to be removed from the mosquito population at each time step. The mosquito's lifespan (*L*_*m*_ for males, *L*_*f*_ for females) needs to be considered, with mosquitoes that die naturally being removed from the population.

(7) $$\begin{array}{l} N_{a_{t+1}}=N_{a_{t}}\left(1-P_{ma}\right) \end{array}$$

In the population, a fraction of the total female mosquitoes is assumed to produce new larvae. Since each pair of parents were randomly selected, an ER random graph model (Barabasi and Albert, 1999) was applied to simulate the mating process:

1. Consider the mosquito set (female mosquitoes are in the fraction),
2. Randomly select a female mosquito that has not mated in the current time step and not reached the maximum number of times eggs can be laid and a random male mosquito from the sets,
3. Produce new larvae,
4. Go to (2) until all the female mosquitoes in the fraction have been selected,
5. End.

The model considers the female mosquitoes' fertility ability (i.e., the maximum number of times that larvae can be produced). The genotypes of the new larvae are determined as follows:

(8) $$\begin{array}{l} N_{nl,z}\;=b{\cdot N}_{f,x}{\cdot N}_{egg}\cdot \frac{N_{m,y}}{N_{m}}\cdot P\left(z|x,y\right) \end{array}$$

(9) $$\begin{array}{l} N_{nl}\;=\sum_{z}N_{nl,z}, \end{array}$$

where, *N*_*nl, z*_ is the number of new larvae for genotype *z, b* is the daily fertility rate of a female mosquito, *N*_*egg*_ is the number of eggs one female can lay at each time step, *N*_*f, x*_ is the number of female mosquitoes in a fraction for genotype *x* that can reproduce (a female mosquito cannot lay eggs more than three times within its lifespan), *N*_*m, y*_ is the number of male mosquitoes for genotype *y*, *N*_*m*_ is the number of male mosquitoes, *P*(*z*|*x, y*) is the probability of new larvae with genotypes *x* and *y* having *z* (*z* is the larval genotype, see Table 1), *N*_*nl*_ is the total number of new larvae, $N_{f,x}\frac{N_{m,y}}{N_{m}}$ represents the probability of a female mosquito with genotype *x* mating with a male mosquito of genotype *y*. As mentioned in the larva section, the offspring may possess different genotypes (Table 1).

**Table 1:** Larval genotype outcomes based on parental genotypes: SS, RS, RR.

**Female genotype**	**Male genotype**	**New larvae genotype**
SS	SS	100% SS
SS	RS	50% SS, 50% RS
SS	RR	100% RS
RS	SS	50% SS, 50% RS
RS	RS	25% SS, 50% RS, 25% RR
RS	RR	50% RS, 50% RR
RR	SS	SS 100% RS
RR	RS	50% RS, 50% RR
RR	RR	100% RR

Considering that only female mosquitoes consume blood meals (Chen, 2011), there are four situations for when a female mosquito bites a human: an uninfected mosquito bites an uninfected human, an infected mosquito bites an infected human, an infected mosquito bites an uninfected human, or an uninfected mosquito bites an infected human (Aleksejs et al., 2015). This model only considers the last two situations since recurrent blood meals from infected humans are assumed not to have any impact on the incubation of the disease. Once an infected mosquito bites an uninfected person, there is a probability that the person becomes infected (*P*_*hi*_) and a probability of remaining healthy (1 – *P*_*hi*_). An infected person then enters the incubation stage, which will last for *N*_*inc*_ time steps. When an uninfected mosquito bites an infected human, the probability of an SS mosquito becoming infected is defined as *P*_*ssi*_, whereas the probability of infection for an RS genotype is defined as *P*_*rsi*_ f. Mosquitoes have no incubation stage. In humans, a person is unable to transmit malaria to a biting mosquito during the incubation stage. When the incubation period passes, the person becomes malaria-positive and can infect mosquitoes, but after *N*_*rec*_, the person will recover from malaria. Recurrent instances of an infected mosquito biting an infected human are assumed not to have any impact on the state. Furthermore, the number of mosquitoes is assumed to be linear with the number of people at the location since female mosquitoes rely on people for blood meals.

### Transmission between populations

Both humans and mosquitoes may migrate between mixed populations, with human mobility potentially affecting malaria prevalence between populations. Thus, it is important to simulate this dynamic. Mosquito migration can not only introduce malaria to an uninfected population, but also propagate refractory genes.

### Human mobility

Human travel, human migration, or other movements between populations are mainly assumed to be map-based and reliant on the roads between the communities (Liu et al., 2011). In the map, each community is a node, and each road is an edge. This model does not consider other modes of transportation, such as airplanes or trains, because they are not the main ways that people move between communities. The number of people moved to another node is based on the weight of the edge between the adjacent nodes as follows:

(10) $$\begin{array}{l} N_{m,t,h}\;={\;N}_{t,h}\cdot P_{m,h} \end{array}$$

(11) $$\begin{array}{l} N_{u,v,h}\;={\;N}_{m,t,h}\cdot \frac{w_{u,v}}{\sum_{i}w_{u,i}} \end{array}$$

where, *N*_*m, t, h*_ is the number of people moving to all the adjacent nodes, *N*_*t, h*_ is the number of people in the population, *P*_*m, h*_ is the probability that people will move, *N*_*u, v, h*_ is the number of people moving from node *u* to node *v*, and *w*_*u, v*_ is the weight (i.e., the distance of the road connecting *u* and *v*).

### Mosquito mobility

Mosquito movement is not road based but map based, with mosquitoes naturally moving within a maximal range (*D*_*mf*_) of their original habitat (Kaufmann and Briegel, 2004). Hence, if there are two locations within *D*_*mf*_, those nodes can be connected as follows:

(12) $$\begin{array}{l} N_{m,t,m}\;={\;N}_{t,m}\cdot P_{m,m} \end{array}$$

(13) $$\begin{array}{l} N_{u,v,m}\;=N_{m,t,m}\cdot \frac{D_{u,v}}{\sum_{i}D_{u,i}}, \end{array}$$

where, *D*_*u, v*_ is the distance (<10 km) from nodes *u* to *v*.

### Map network simplification and visualization

*Anopheles gambiae* is the main mosquito that transmits malaria in Africa. Herein, a map network was created using an OpenStreetMap (OSM, https://www.openstreetmap.org/) dataset instead of using a network generation model. Since this model focuses on modeling spatial disease dynamics, it is important for the map to accurately represent the actual topology. Although the obtained OSM data depict road structures rather than connectivity, it is still important to describe the connectivity between two nodes. The end nodes of each road are assumed to be the original nodes. However, it was found that the nodes with *deg* (*v*) = 2 (*deg* (*v*), it can be defined as the number of roads that the node *v* connects with are just the joint nodes and should be removed. Since the edges are weighted, the new weight of the edge will be the sum of the weight of the removed edges. The edge weight between the nodes still captures the actual road length; the simplification reduces the number of nodes and edges and makes the network easier to handle. The aim is that the nodes in the map network should represent settlements, whereas the edges represent the direct connectivity between those settlements.

Although the complicated road structures are present in the OSM map data, they include much noisy data and only a small amount of information marked as villages; hence, the settlement structures could be extracted from the map data using PTV and Gephi. It is assumed that the settlement is often a densely connected cluster that is similar to a community within a social network. Thus, a community finding method is used to detect the communities in the network. There are some typical community detection methods such as fast unfolding of community hierarchies (Blondel et al., 2008), hierarchical agglomeration algorithm (Clauset et al., 2004), maps of information flow methods (Rosvall and Bergstrom, 2008), using the eigenvectors of matrices methods (Newman, 2006), fast unfolding of communities (Blondel et al., 2008), evaluating the community structure (Newman and Girvan, 2004), and near linear time algorithm (Raghavan et al., 2007), which can be evaluated by a real network (if it exists) or modularity. As there is no ground truth data that can map the road network nodes to the settlements, modularity is the only option. Modularity compares the number of edges within a cluster to that of a random partitioning and is defined as

(14) $$Q\;=\sum_{i\;=\;1}^{L}(e_{ii-}a_{i}^{2})\;$$

where, *L* is the number of communities, *e*_*ii*_ is the edge density in the *i*th community, and $a_{i}^{2}$ is the expected value in a random network (Watada et al., 2012). The BGLL algorithm is then used to reveal the hierarchical community structure of the networks and has been shown to outperform many other known community detection methods (Blondel et al., 2008) and is appropriate given that the simplified road network is weighted.

**Figure 1:**
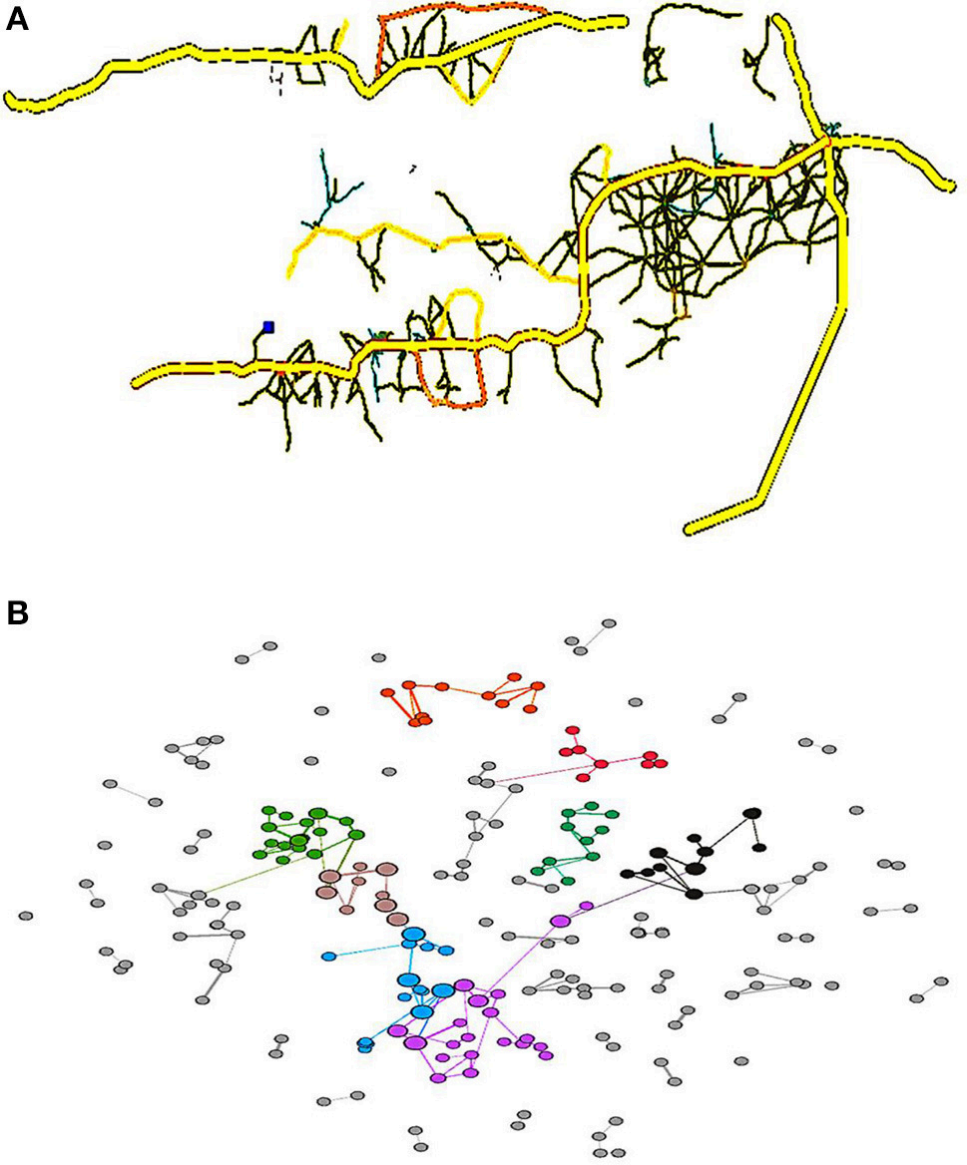
Road map of the main districts in Gambia **(A)** and a corresponding road network **(B)**. Each node is colored according to the community to which it is assigned, with commonly colored nodes typically located nearby and densely clustered. This means that the assumption that complicated road structures are present within a settlement is likely true. The node sizes reflect the betweenness values.

The original OSM data needs to be simplified and converted for visualization using PTV Visum and Gephi. The PTV Visum provides urban road visualization and converts the OSM map data into a Gephi data format, thus enabling network visualization and corresponding metrics to be computed. In addition, Gephi readily implements the BGLL algorithm. This implementation supports community detection for weighted graphsand represents the geographical distance between the nodes and the clusters nearby nodes. The original road networks within the main districts in Gambia (PTV Visum) were then converted into a simplified network (Gephi) to generate a map (Figure 1), with the network nodes being colored according to their assigned communities.

In summary, the simulation included two sections for each time step: evolution within the populations and transmission between the populations. The first section includes death (adult mosquitoes and larvae), mating (adult mosquitoes), reproduction (new larvae), and biting (female adult mosquitoes and humans). In this section, a new mosquito population with a new infection status (mosquitoes and humans) will be produced. In the second section, mosquito and human mobility are examined on the basis of the distance and the map. In this section, mosquito and human populations within some nodes will change.

## Results

### Parameters

The parameters used for this model are based on previous findings (Boëte and Koella, 2002; Kaufmann and Briegel, 2004; Brasil et al., 2011; Chen, 2011; Zhong and Yan, 2011; CDC, 2012; Johnston et al., 2013; Aleksejs et al., 2015) or were estimated (Table 2). Notably, some of the values are approximations or estimations. For example, the malaria incubation period in mosquitoes depends upon the climate and season. If these data are known, the parameters should be adjusted to the specific region in question.

**Table 2:** The parameters used for the model.

**Parameter**	**Interpretation**	**Initial or default value**	**Source**
*d*	Larval death rate	0.2	Boëte and Koella, 2002
*r*	Prevalence of malaria	0.5	Aleksejs et al., 2015
*s*	Resistance to malaria	0.9	Aleksejs et al., 2015
*h*	Dominance	1	Boëte and Koella, 2002
α	Parasite virulence	0.3	Aleksejs et al., 2015
*k*	Daily probability of mosquito biting	0.9	Aleksejs et al., 2015
ε	Tune parameter	< 0.001	Decided
*P_*ma*_*	Daily probability of mosquito mortality	0.195	CDC, 2012
*b*	Daily fertility rate	0.58	Aleksejs et al., 2015
*N_*egg*_*	Number of laying eggs for each female mosquito at each time step	200	Chen, 2011
*P_*rg*_*	Maximum times for pregnant	3	Chen, 2011
*P_*hi*_*	Human infection probability	1	Aleksejs et al., 2015
*P_*ssi*_*	SS mosquito infection probability from biting	1	Aleksejs et al., 2015
*P_*rsi*_*	RS mosquito infection probability from biting	1	Aleksejs et al., 2015
*N_*inc*_*	Incubation period in humans	12	Brasil et al., 2011
*N_*rec*_*	Recovery period for humans	32	Johnston et al., 2013
*L_*m*_*	Male mosquito lifespan	17	Zhong and Yan, 2011
*L_*f*_*	Female mosquito lifespan	23	Zhong and Yan, 2011
*P_*m, h*_*	Daily human migration probability	0.2	Aleksejs et al., 2015
*P_*m, m*_*	Daily mosquito distance migration probability	0.1	Aleksejs et al., 2015
*D_*mf*_*	Maximal mosquito migration distance	10 km	Kaufmann and Briegel, 2004

*The majority of the parameters were previously described or they were estimated. While this should be adjusted if there are known cases of refractory mosquitoes being released to the wild, this is not possible in the short term*.

## Malaria-control strategies

In this study, an evolution model and mobility network were used to perform simulations that predict how the initial percentage of refractoriness is affected by different deployment strategies and how the refractoriness propagates throughout the network. The simulations included the following: (1) deploying different ratios of refractory mosquitoes evenly in every location; (2) deploying the refractory mosquitoes in one random location; (3) deploying the refractory mosquitoes in the location with the largest betweenness value based on centrality (Coulombe-Huntington and Xia, 2017); (4) deploying the refractory mosquitoes evenly in random locations; or (5) deploying the refractory mosquitoes in some locations based on their betweenness values. The refractory mosquito population size was limited on the basis of the cost-effective factor. Each simulation was performed for 360 days (360 time steps/1 year) and plotted in 30 (1 month), 60 (2 months), 90 (3 months), and 180 time steps (half a year). tOwing to the short lifespan of mosquitoes, performing the simulation for more than 1 year was not necessary.

The main districts in Gambia include the North Bank, West Coast, and Lower River (longitude: −16.3628 to −15.8670, latitude: from 13.4684 to 13.2600; total area: 1681 km^2^; estimated human population: 216,000), with these areas being incorporated into the mobility network simulations, and a total of 108 nodes were identified after the simplification. The mosquito population size was set to 2,160,000, which was 10 times that of the human population size (Sun and Zhu, 2010). In addition, the initial malaria prevalence in the human population was 50%.

### Refractory population ratio and the prevalence of refractoriness and malaria

**Figure 2:**
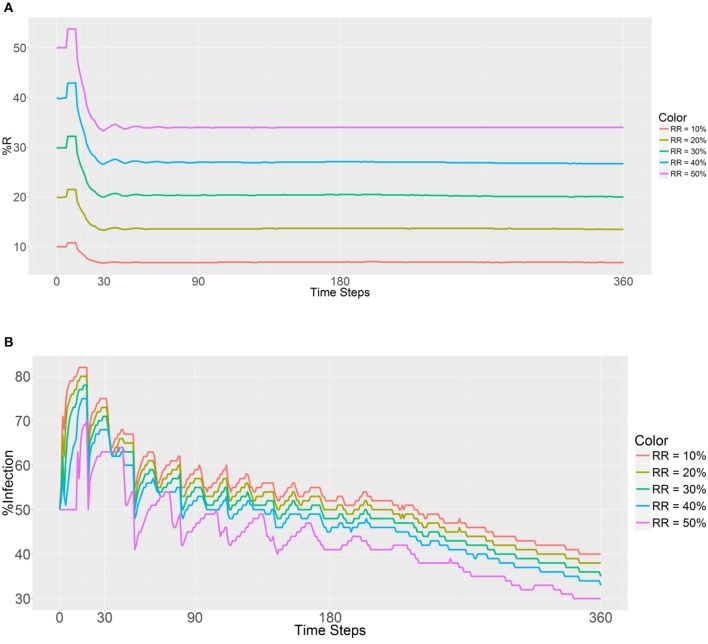
Prevalence of the R allele **(A)** and malaria **(B)** after 1 year. Populations received refractory mosquitoes, making the RR genotype comprise 10, 20, 30, 40, or 50% of the population. “RR = 10%” refers to refractory mosquitoes (RR) being deployed to make up 10% of the total population. The values are plotted in 0, 30, 90, 180, and 360 time steps.

The introduced RR population (refractory mosquitoes) did not exceed the SS population, with the initial RR:total population ratios ranging from 0.1 to 0.5 in every location. The step for the independent variables was 0.1. For this simulation, the RR populations within each human population were equal to evaluate the prevalence of the R allele after 30, 90, 180, and 360 days. The first plot (Figure 2A) shows that the introduction of a larger number of RR mosquitoes has a significantly positive impact on the prevalence of the R allele and promotes an increase in the RR genotype (larger sizes of initial RR populations produced higher R prevalence values, it becomes more pronounced as the initial RR ratio increases). Although this trend is observed across all the time steps (30, 90, 180, and 360 days) postdeployment, there are obvious sudden decreases at the beginning across all the initial RR ratios. Once the female RR mosquitoes reach their fertility ability (i.e., the maximum number of times eggs can be laid, Table 1), there is a sudden decrease in the prevalence of the R allele and then the curve displays a plateau once the gene pool stabilizes. We note that the decrease does not affect the long-term trend. The random factors in the model account for a few small mutations during the process. Furthermore, the R prevalence value was found to be below the initial deployment level during all of the time steps.

As expected, a higher initial number of RR mosquitoes lowered the malaria prevalence values in malaria-positive and malaria-negative humans (Figure 2B). Furthermore, a gradual decrease in malaria infection was noted across all the RR ratios during the entire process. Thus, it seems that a higher prevalence of the refractory R allele will reduce the prevalence of malaria as expected. In addition, the initial malaria prevalence after the introduction of 10,000 refractory mosquitoes (50% of the total) to each population did not change until 11 time steps had been reached. The reason for this finding is most likely attributable to the fact that refractory female mosquitoes (RR) cannot be infected by a person until the new female larvae reach adulthood.

### Different deployment strategies and malaria prevalence

In the earlier-mentioned simulations, the impact of the RR population size ratio on the prevalence of the refractory R allele was examined, with refractory mosquitoes being assumed to be distributed across all the 108 populations, which would lead to a high deployment cost. To lower the cost, it would be possible to introduce refractory populations to a subset of nodes. In network science, betweenness is an important metric of a node's centrality and captures the number of shortest paths for each pair of nodes and chooses the nodes for mosquito migration. This ensures that the refractory mosquitoes are deployed in the populations that will promote a faster spread rather than randomly selecting populations.

**Figure 3:**
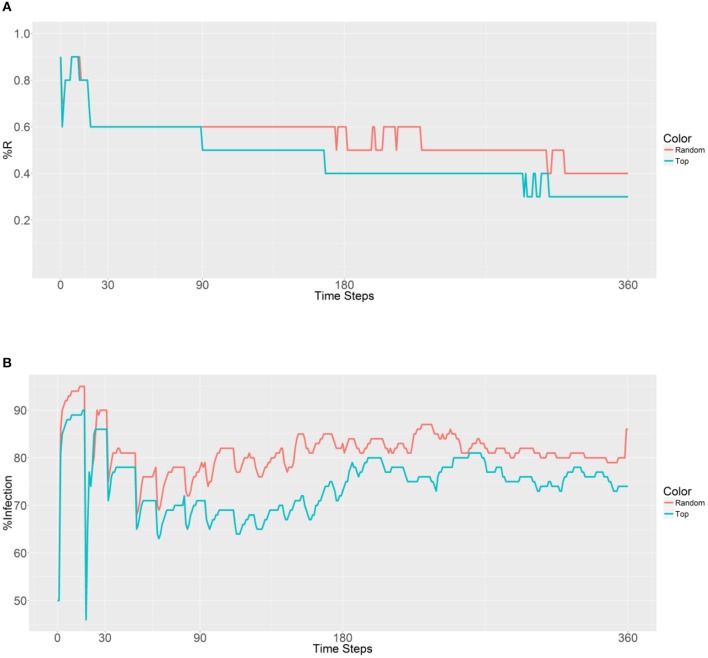
Prevalence of the R allele **(A)** and malaria **(B)** 1 year after deploying 10,000 refractory mosquitoes into a randomly selected population or a top population based on the betweenness value.

In these simulations, all of the nodes were ranked on the basis of their betweenness values, and 10,000 refractory mosquitoes were deployed. These mosquitoes were deployed using two strategies—random selection (the first strategy) and top nodes selected based on betweenness value (the second strategy)—with 1, 5, 10, 20, or 40 nodes (betweenness value based) selected, and the prevalence of the R allele and malaria were observed (Figure 3). In the first plot (Figure 3A), both the curves have similar trends until 90 time steps, and then the first strategy shows a lower R allele prevalence. These findings suggest that the R allele spreads more slowly when using the first strategy and leads to a disappearance of the R allele over time within a population.

When examining the second plot (Figure 3B), both the curves have two obvious sudden increases and one sudden decrease before 30 time steps. The first increase is associated with the beginning of a large-scale infection, with the initial malaria period continuing until 13 time steps. The recovery period was reached after 19 time steps and accompanied by a decrease and sudden increase until a plateau was reached. The second strategy results in a lower malaria prevalence (between 60 and 80% in most time steps) when compared to the first strategy (between 70% and 90% in most time steps). However, both the strategies do not reduce the levels below the initial malaria level (50%). It seems that the introduction of the refractory 10,000 mosquitoes into a single population has no effect on the reduction of malaria because of the slow spreading of the R allele throughout the population.

**Figure 4:**
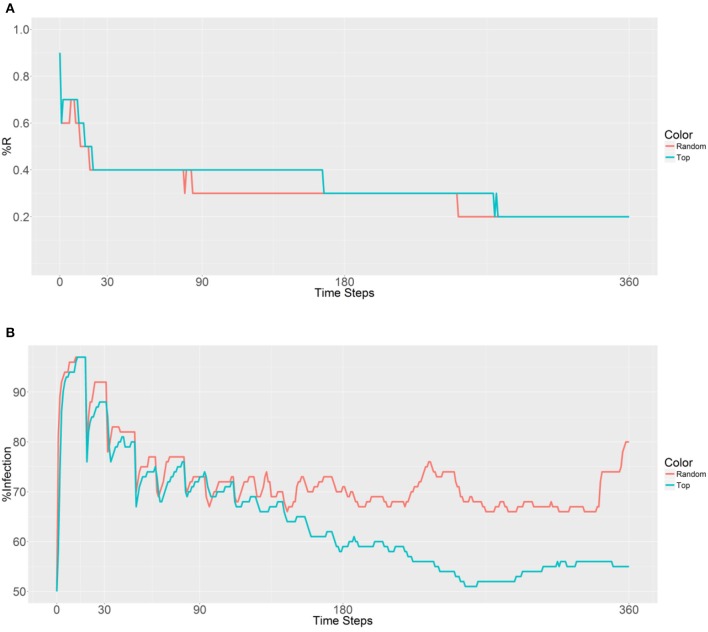
Prevalence of the R allele **(A)** and malaria **(B)** 1 year after deploying 10,000 refractory mosquitoes into 5 randomly selected or 5 top betweenness value-based populations (*n* = 2,000 per population).

When considering the deployment of 10,000 refractory mosquitoes into 5 populations (2,000 per population) using random selection or the top 5 betweenness value-based approaches, the same R allele prevalence was observed in the first plot (Figure 4A). Both the strategies show a gradual decrease due to fertility limits of female mosquitoes and then the curves stabilize. As expected, curve stabilization with the first strategy was reached earlier than with the second strategy. However, in the second plot that examined malaria prevalence, different trends were observed (Figure 4B). The first curve gradually increases, while the second curve decreases after 153 time steps. We note that when using the second strategy, the malaria prevalence gradually decreases and achieves a lower value than the original prevalence (50%) after 251 time steps. These findings suggest that deployment within the top 5 betweenness value-based populations (second strategy) has a significant effect on the spreading of the R allele and reduces malaria prevalence. However, it does not reduce the original number of infected humans (50% of malaria prevalence).

**Figure 5:**
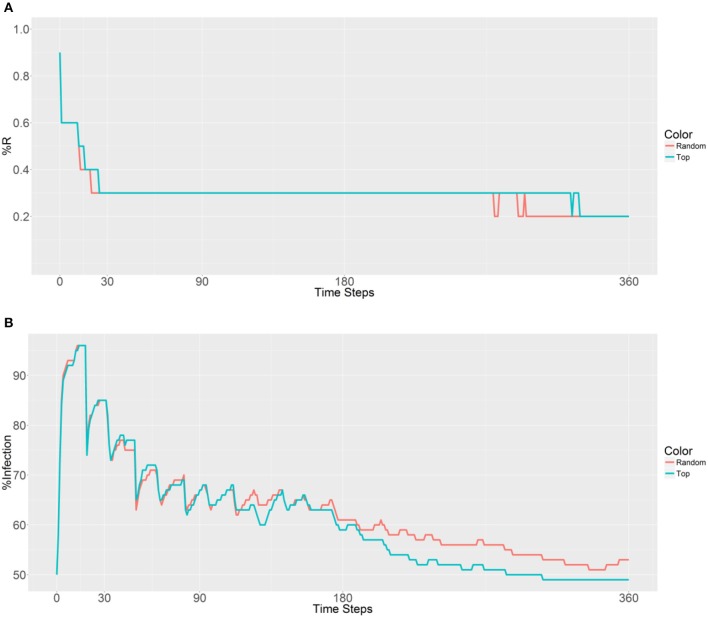
Prevalence of the R allele **(A)** and malaria **(B)** 1 year after deploying 10,000 refractory mosquitoes into 10 randomly selected or 10 top betweenness value-based populations (*n* = 1,000 per population).

A similar outcome is observed in Figure 5, with the first strategy reaching its lowest value earlier than the second strategy because of the rapid spreading of R allele in the second strategy. When examining malaria prevalence (Figure 5B), the two curves have similar trends and gradually decrease as the time steps increase. However, a lower value (49%) is achieved by using the second strategy when compared to the original value (50%) after 306 time steps. Thus, it seems that deployment in the top 10 populations can reduce the initial malaria levels.

**Figure 6:**
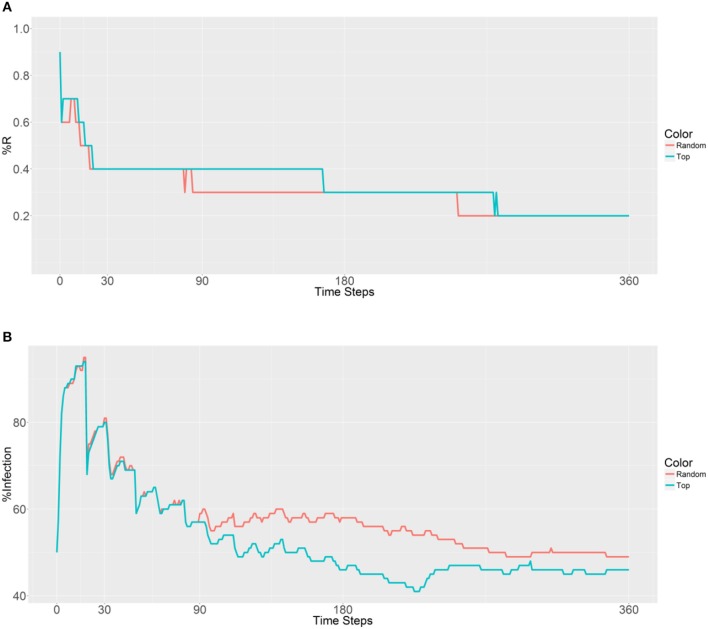
Prevalence of the R allele **(A)** and malaria **(B)** 1 year after deploying 10,000 refractory mosquitoes into 20 randomly selected or 20 top betweenness value-based populations (*n* = 500 per population).

**Figure 7:**
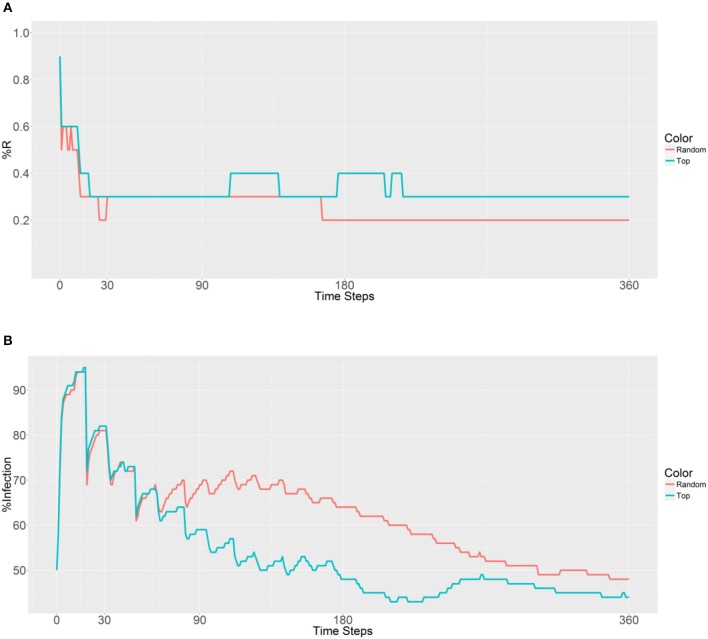
Prevalence of the R allele **(A)** and malaria **(B)** 1 year after deploying 10,000 refractory mosquitoes into 40 randomly selected or 40 top betweenness value-based populations (*n* = 250 per population).

Next, 10,000 refractory mosquitoes were deployed among 20 populations, and a similar R allele prevalence was observed when using either strategy (Figure 6). However, some lower values were observed using the second strategy, with under 50% infected from 158 to 360 time steps and a value of 41% obtained from 225 to 228 time steps (Figure 6B). Thus, the use of the second strategy based on betweenness had a significant effect on the reduction of the malaria prevalence. However, when deploying the 10,000 refectory mosquitoes among 40 populations, the same R allele prevalence was observed, but the malaria prevalence did not become as low as it did when using 20 populations only (Figures 6, 7). Thus, increasing the distribution to 40 populations, when using the second strategy, did not further reduce the malaria prevalence or offer any benefit over deploying only 20 populations.

### Probabilistic uncertainty

**Figure 8:**
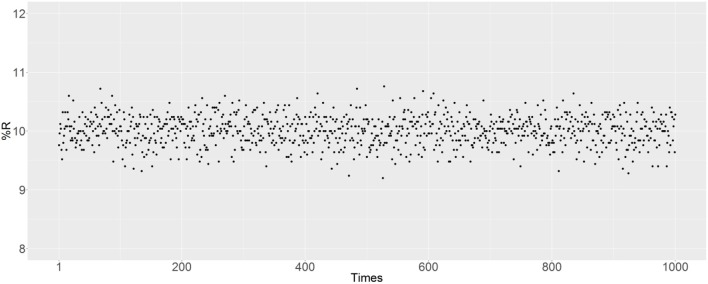
Prevalence of the R allele after simulating 1,000 independent matings. The parents were randomly selected after removing the set.seed () function. The initial RR ratio was 10%, and the initial male-to-female ratio was 1:1; thus 20% of female mosquitoes have an RR genotype.

There are some random factors in the simulation, with the main probabilistic uncertainty being the mating of randomly selected male and female mosquitoes. In the R script, the set.seed () function was used to set the random seed to simulate reproduction. The simulation was also performed to evaluate the effect of probabilistic uncertainty on the results. In Figure 8, 1,000 independent matings are examined after removing the set.seed () function, with an initial R prevalence of 10% and the parents are randomly selected. The R prevalence values were found to always be around 10%, and the probabilistic uncertainty does not have a significant impact on the results.

## Discussion

This study proposed a method to create a metanetwork on the basis of the geographic map data of Gambia and constructed a model to simulate the evolution within a mosquito population and transmission between populations. Furthermore, a series of simulations were performed to evaluate different deployment strategies for the reduction of the prevalence of malaria.

The map network in a specific area is essential for the simulations. The main districts in Gambia were chosen as the map example since *A. gambiae* is the main mosquito that transmits malaria in Africa. The map data were downloaded from OMS and transformed into the Gephi format for visualization and measurements. This approach can be used to produce a realistic mobility network for other regions and can also be used for other mosquito-borne diseases, such as dengue or yellow fever. Herein, an evolutionary model was implemented to evaluate the genetic control measures for malaria, including birth, death, reproduction, bite, infection, incubation, recovery, and transmission. The final model combines two components: the evolution within a population and migration between populations.

The first simulation shows that the introduction of the same number of refractory mosquitoes into each population can increase the prevalence of the RR genotype and the R allele and can lower malaria prevalence. Considering the cost of deployment, 10,000 refractory mosquitoes were deployed among 1, 5, 10, 20, or 40 populations, with populations being selected by using a random selection approach or choosing populations with top betweenness values. When only deploying the mosquitoes between 1 and 5 nodes, no benefit was noted. However, deployment among 10, 20, or 40 populations did effectively reduce malaria prevalence, with a distribution between 20 populations being optimal.

It seems that there is a negative relationship between R allele prevalence and malaria prevalence when deploying refractory mosquitoes in a small set of populations. However, there seems to be no advantage in the introduction of the refractory mosquitoes into a big fraction of central populations. Moreover, the introduction of the refractory mosquitoes into a small fraction of central populations can enhance the spread of the refractory gene throughout the populations and can reduce malaria. Furthermore, a simulation was performed to evaluate the impact of the probabilistic uncertainty of mating on the simulation, and shows that the random factors had no significant impact on the results.

In this work, since some factors or activities are based on one day such as human mobility and mosquito lifespan, we chose one day as the time step. We can also choose another time step if new evidence is available.

We did not use other approaches such as the Bayesian approach (Fernández et al., 2013); the reasons are as follows: (1) we did not have the real dataset, so we used the parameters value in the references; (2) we decided not to introduce more probabilistic uncertainty factors, and our work focused on transmission-control strategies. We also used the set.seed() function in the R script to ensure that we reproduced the results; and (3) we will definitely introduce more random factors, if we have the real dataset in future.

## Conclusions

Malaria is an infectious disease that is caused by a parasitic plasmodium. Many studies have attempted to counter malaria by developing genetically modified mosquitoes. The aim of this study was to create a model that simulates the transmission of malaria resistance among populations. The results show that the deployment of a higher RR mosquito ratio can lower malaria prevalence. When trying to determine a cost-effective deployment strategy, it was found that deployment among a relatively small fraction of central nodes can spread the R allele and reduce the number of the infected humans gradually. Furthermore, the standard network centrality measurements were found to be suitable for planning deployment locations. Future studies will concentrate on verifying the results presented here and further consider the factors that influence human mobility. If datasets for malaria parasite prevalence in human and mosquito populations were available, the results of the simulation could have been verified. Furthermore, modeling other factors, such as historical sites or economic factors, could be incorporated, but socioeconomic and demographic data would be required.

## Author contributions

BL performed the data analysis, supervised the project, and drafted the manuscript. W-JW and HZ worked on the initial programing of evolution methods. XL provided the crucial concepts. Z-YA provided valuable advice on the project improvement to the manuscript. FZ revised part of the manuscript. All the authors read and approved the final manuscript.

### Conflict of interest statement

The authors declare that the research was conducted in the absence of any commercial or financial relationships that could be construed as a potential conflict of interest.

## References

[B1] Aleksejs S. Simon D. Oscar G. (2015). A Metapopulation Model for Predicting the Success of Genetic Control Measures for Malaria. St. Andrews: University of St Andrews.

[B2] Barabasi A. L. Albert R. (1999). Emergence of scaling in random networks. Science 286, 509–512. 10.1126/science.286.5439.509 10521342

[B3] Benitez J. A. Labra J. E. Quiroga E. Martin V. Garcia I. Marques-Sanchez P.. (2017). A web-based tool for automatic data collection, curation, and visualization of complex healthcare survey studies including social network analysis. Comp. Math. Methods Med. 2017:2579848. 10.1155/2017/2579848 28529537 PMC5424185

[B4] Blondel V. D. Guillaume J. L. Lambiotte R. (2008). Fast unfolding of community hierarchies in large networks. J. Stat. Mech. arXiv:0803.0476. [preprint] 10.1088/1742-5468/2008/10/P10008

[B5] Boëte C. Koella J. C. (2002). A theoretical approach to predicting the success of genetic manipulation of malaria mosquitoes in malaria control. Malar J. 1, 1–7. 10.1186/1475-2875-1-3 12057019 PMC111501

[B6] Brasil P. de Pina Costa A. Pedro R. S. da Silveira Bressan C. da Silva S.. (2011). Unexpectedly long incubation period of Plasmodium vivax malaria, in the absence of chemoprophylaxis, in patients diagnosed outside the transmission area in Brazil. Malaria J. 10:122. 10.1186/1475-2875-10-122 21569554 PMC3120730

[B7] Caravaca M. Sanchez-Andrada P. Soto A. Alajarin M. (2014). The network simulation method: a useful tool for locating the kinetic-thermodynamic switching point in complex kinetic schemes. Phys. Chem. Chem. Phys. 16, 25409–25420. 10.1039/C4CP02079K 25342168

[B8] CDC (2012). Anopheles mosquitoes. Available online at: http://www.cdc.gov/malaria/about/biology/mosquitoes/

[B9] Chen G. F. (2011). Changing the mosquito gene No longer spread the disease. Digest Sci. Tech. 1:6.

[B10] Clauset A. Newman M. E. Moore C. (2004). Finding community structure in very large networks. Phys. Rev. E 70:066111. 10.1103/PhysRevE.70.066111 15697438

[B11] Coulombe-Huntington J. Xia Y. (2017). network centrality analysis in fungi reveals complex regulation of lost and gained genes. PLoS ONE 12:e0169459. 10.1371/journal.pone.0169459 28046110 PMC5207763

[B12] Fernández D. P. Auzmendi J. Peña D. García V. E. Moffatt L. (2013). Bayesian approach to model CD137 signaling in human, M. tuberculosis *in vitro* responses. PLoS ONE 8:e55987 10.1371/journal.pone.0055987 23437083 PMC3577821

[B13] Gasparri A. Meloni S. Panzieri S. (2009). Growing fully distributed robust topologies in a sensor network, in Modelling, Estimation and Control of Networked Complex Systems. (Springer), 143–158.

[B14] Johnston G. L. Smith D. L. Fidock D. A. (2013). Malaria's missing number: calculating the human component of R0 by a within-host mechanistic model of Plasmodium falciparum infection and transmission. PLoS Comput Biol. 9:e1003025. 10.1371/journal.pcbi.1003025 23637586 PMC3630126

[B15] Kaufmann C. Briegel H. (2004). Flight performance of the malaria vectors Anopheles gambiae and Anopheles atroparvus. J. Vect. Ecol. J. Soc. Vect. Ecol. 29, 140–153. 15266751

[B16] Kiang R. Adimi F. Soika V. Nigro J. Singhasivanon P. Sirichaisinthop J.. (2006). Meteorological, environmental remote sensing and neural network analysis of the epidemiology of malaria transmission in Thailand. Geospat Health. 1, 71–84. 10.4081/gh.2006.282 18686233

[B17] Liu J. Yang B. Cheung W. K. Yang G. (2012). Malaria transmission modelling: a network perspective. Infect. Dis. Poverty 1:11. 10.1186/2049-9957-1-11 23849949 PMC3710080

[B18] Liu Y. Xiao Y. Gao S. Kang C. G. Wang Y. L. (2011). Overview of human mobility research based on location aware device. Geograp. Geo-Inform. Sci. 27, 8–13. Available online at: http://www.en.cnki.com.cn/Article_en/CJFDTOTAL-DLGT201104003.htm

[B19] Newman M. E. (2006). Finding community structure using the eigenvectors of matrices. Phys. Rev. E 74:036104 10.1103/PhysRevE.74.036104 17025705

[B20] Newman M. E. Girvan M. (2004). Finding and evaluating community structure in networks. Phys. Rev. E 69:026113. 10.1103/PhysRevE.69.026113 14995526

[B21] Orr H. A. (2009). Fitness and its role in evolutionary genetics. Nat. Rev. Genet. 10, 531–9. 10.1038/nrg2603 19546856 PMC2753274

[B22] OXITEC (2018). The Friendly Mosquitoes are a Targeted Vector-Control Solution to Pest Mosquitoes That Spread Disease. Available online at: http://www.oxitec.com/

[B23] Ozkanlar A. Clark A. E. (2014). ChemNetworks: a complex network analysis tool for chemical systems. J. Comput. Chem. 35, 495–505. 10.1002/jcc.23506 24311311

[B24] Raghavan U. N. Albert R. Kumara S. (2007). Near linear time algorithm to detect community structures in large-scale networks. Phys. Rev. E Stat. 76:036106. 10.1103/PhysRevE.76.036106 17930305

[B25] Rosvall M. Bergstrom C. T. (2008). Maps of information flow reveal commu-nity structure in complex networks. PNAS 105:118 10.1073/pnas.0706851105 18172203 PMC2224170

[B26] Rothkegel A. Lehnertz K. Conedy A. (2012). Scientific tool to investigate complex network dynamics. Chaos 22, 013125. 10.1063/1.3685527 22463001

[B27] Scott T. W. Takken W. Knols B. G. J. Boete C. (2002). The ecology of genetically modified mosquitoes. Science 298:5591. 10.1126/science.298.5591.117 12364785

[B28] Small M. Tse C. K. (2010). Complex network models of disease propagation: modelling, predicting and assessing the transmission of SARS. Hong Kong Med. J. 16, 43–4. 20864748

[B29] Steele Gray C. Wodchis W. P. Upshur R. Cott C. McKinstry B. Mercer S.. (2016). Supporting goal-oriented primary health care for seniors with complex care needs using mobile technology: evaluation and implementation of the health system performance research network, bridgepoint electronic patient reported outcome tool. JMIR Res. Prot. 5:e126. 10.2196/resprot.5756 27341765 PMC4938886

[B30] Steinig E. J. Neuditschko M. Khatkar M. S. Raadsma H. W. Zenger K. R. (2016). Netview p: a network visualization tool to unravel complex population structure using genome-wide SNPs. Mol. Ecol. Resourc. 16, 216–227. 10.1111/1755-0998.12442 26129944

[B31] Sun Y. Zhu Z. W. (2010). Surveillance of Vector Mosquitoes of Malaria and Its Related Environmental Factors in Wau, Sudan. J. Acta Parasitol. et Med. Entomol. Sinica. 1, 24–28. 10.1186/1475-2875-8-268

[B32] Tapanelli S. Habluetzel A. Pellei M. Marchio L. Tombesi A. Cappare A.. (2017). Novel metalloantimalarials: Transmission blocking effects of water soluble Cu, Ag(I) and Au(I) phosphane complexes on the murine malaria parasite Plasmodium berghei. J. Inorgan. Biochem. 166, 1–4. 10.1016/j.jinorgbio.2016.10.004 27815977

[B33] Tasai S. Saiwichai T. Kaewthamasorn M. Tiawsirisup S. Buddhirakkul P. Chaichalotornkul S.. (2017). Artesunate-tafenoquine combination therapy promotes clearance and abrogates transmission of the avian malaria parasite Plasmodium gallinaceum. Vet. Parasitol. 233, 97–106. 10.1016/j.vetpar.2016.12.008 28043395

[B34] Taylor B. J. Lanke K. Banman S. L. Morlais I. Morin M. J. Bousema T.. (2017). A direct from blood reverse transcriptase polymerase chain reaction assay for monitoring falciparum malaria parasite transmission in elimination settings. Am. J. Trop. Med. Hygiene 97, 533–543. 10.4269/ajtmh.17-0039 28722583 PMC5544097

[B35] Teklemariam M. Assefa A. Kassa M. Mohammed H. Mamo H. (2017). Therapeutic efficacy of artemether-lumefantrine against uncomplicated Plasmodium falciparum malaria in a high-transmission area in northwest Ethiopia. PLoS ONE 12:e0176004. 10.1371/journal.pone.0176004 28445503 PMC5405980

[B36] Telesford Q. K. Simpson S. L. Burdette J. H. Hayasaka S. Laurienti P. J. (2011). The brain as a complex system: using network science as a tool for understanding the brain. Brain Connect. 1, 295–308. 10.1089/brain.2011.0055 22432419 PMC3621511

[B37] The Earth Institute of Columbia University (2001). A New Global Commitment to Disease Control in Africa. 10.1038/87830 11329042

[B38] Theisen M. Jore M. M. Sauerwein R. (2017). Towards clinical development of a Pfs48/45-based transmission blocking malaria vaccine. Exp. Rev. Vacc. 16, 329–336. 10.1080/14760584.2017.1276833 28043178

[B39] Toure M. Petersen P. T. Bathily S. N. Sanogo D. Wang C. W. Schioler K. L.. (2017). Molecular evidence of malaria and zoonotic diseases among rapid diagnostic test-negative febrile patients in low-transmission season, Mali. Am. J. Trop. Med. Hygi. 96, 335–337. 10.4269/ajtmh.16-0700 27821696 PMC5303032

[B40] Tucker L. J. M. Vittor A. Rifai S. Valle D. (2017). Does deforestation promote or inhibit malaria transmission in the Amazon? A systematic literature review and critical appraisal of current evidence. Philos. Trans. R. Soc. Lond. Ser. B Biol. Sci. 372:20160125. 10.1098/rstb.2016.0125 28438914 PMC5413873

[B41] Wang X. Zhao X. Q. (2017). A malaria transmission model with temperature-dependent incubation period. Bull. Math. Biol. 79, 1155–1182. 10.1007/s11538-017-0276-3 28389985

[B42] Wanja E. Achilla R. Obare P. Adeny R. Moseti C. Otieno V.. (2017). Evaluation of a laboratory quality assurance pilot programme for malaria diagnostics in low-transmission areas of Kenya (2013). Malaria J. 16:221. 10.1186/s12936-017-1856-2 28545579 PMC5445328

[B43] Watada J. Watanabe T. Phillips W. G. Howlett R. J. Jain L. C. (2012). Intelligent Decision Technologies, in Proceedings of the 4th International Conference on Intelligent Decision Technologies Vol 2 Gifu: Springer Science and Business Media.

[B44] Wilson M. L. Krogstad D. J. Arinaitwe E. Arevalo-Herrera M. Chery L. Ferreira M. U.. (2015). Urban Malaria: understanding its epidemiology, ecology, and transmission across seven diverse ICEMR network sites. Am. J. Trop. Med. Hygi. 93, 110–123. 10.4269/ajtmh.14-0834 26259941 PMC4574269

[B45] World Health Organization (2018). Malaria. Available online at: http://www.who.int/mediacentre/factsheets/fs094/en/

[B46] Yang L. Neale B. M. Liu L. Lee S. H. Wray N. R. Ji N. (2013). Polygenic transmission and complex neuro developmental network for attention deficit hyperactivity disorder: genome-wide association study of both common and rare variants. Am. J. Med. Genet. 162, 419–430. 10.1002/ajmg.b.32169 PMC4321789 23728934

[B47] Ya-umphan P. Cerqueira D. Parker D. M. Cottrell G. Poinsignon A. Remoue F.. (2017). Use of an anopheles salivary biomarker to assess malaria transmission risk along the Thailand-Myanmar border. J. Infect. Dis. 215, 396–404. 10.1093/infdis/jiw543 27932615 PMC5853934

[B48] Zheng W. Liu F. He Y. Liu Q. Humphreys G. B. Tsuboi T.. (2017). Functional characterization of Plasmodium berghei PSOP25 during ookinete development and as a malaria transmission-blocking vaccine candidate. Parasites Vect. 10:8. 10.1186/s13071-016-1932-4 28057055 PMC5217559

[B49] Zhong G. M. Yan Y. (2011). Experimental observation on the Fecundity and longevity of Anopheles sinensis. Chinese J. Vect. Biol. Contr. 11, 265–267. Available online at: http://www.bmsw.net.cn/EN/abstract/abstract10821.shtml

